# Risks in the design of regional hydrogen hub systems: A review and commentary

**DOI:** 10.1111/risa.17449

**Published:** 2024-08-24

**Authors:** Valerie J. Karplus, Ioana Iacob, Emily J. Moore, M. Granger Morgan

**Affiliations:** ^1^ Department of Engineering and Public Policy Carnegie Mellon University Pittsburgh Pennsylvania USA; ^2^ Wilton E. Scott Institute for Energy Innovation Carnegie Mellon University Pittsburgh Pennsylvania USA

**Keywords:** Climate change mitigation, decarbonization, energy transition, hydrogen

## Abstract

Early investments in regional hydrogen systems carry two distinct types of risk: (1) economic risk that projects will not be financially viable, resulting in stranded capital, and (2) environmental risk that projects will not deliver deep reductions in greenhouse gas emissions and through leaks, perhaps even contribute to climate change. This article systematically reviews the literature and performs analysis to describe both types of risk in the context of recent efforts in the United States and worldwide to support the development of “hydrogen hubs” or regional systems of hydrogen production and use. We review estimates of hydrogen production costs and projections of how future costs are likely to change over time for different production routes, environmental impacts related to hydrogen and methane leaks, and the availability and effectiveness of carbon capture and sequestration. Finally, we consider system‐wide risks associated with evolving regional industrial structures, including job displacement and underinvestment in shared components, such as refueling. We conclude by suggesting a set of design principles that should be applied in developing early hydrogen hubs if they are to be a successful step toward creating a decarbonized energy system.

## 1 INTRODUCTION

Deep reductions in the emissions of greenhouse gas (GHG) emissions from energy systems are needed to limit what is already serious, and may soon become catastrophic, climate change. Hydrogen has been proposed as an energy carrier that could be used as one component of a portfolio of solutions to decarbonize energy systems. Today, hydrogen is widely used as an input in a variety of industrial processes; however, hydrogen could play an expanded role in decarbonizing industrial processes in the future (International Energy Agency, 2019a; World Energy Council, 2021).

As of 2024, 60 countries around the world have published national hydrogen road maps (Corbeau & Kaswiyanto, 2024). Most national strategies include their major goals and drivers (primarily decarbonization, diversifying energy supplies, and fostering economic growth); the most commonly employed policy tools include direct financial support, financial incentives, legislative measures, international partnerships, and R&D initiatives (World Energy Council, 2021).

In “The Future of Hydrogen,” the International Energy Agency proposed a similar set of policy actions to encourage a hydrogen economy, including establishing “long‐term signals to foster investor confidence,” bolstering demand from several sectors, mitigating risks along the value chain, encouraging R&D, and setting standards that would remove barriers within the markets (International Energy Agency, 2019b). In fact, of the 60 published national hydrogen roadmaps, 14 explicitly refer to the creation of these colocated systems with terms such as “hydrogen valleys,” “hydrogen ecosystems,” “hydrogen hubs,” or “production poles” (Iacob & Morgan, 2024). Both Northern Europe and the United States have launched publicly funded efforts to encourage the development of “hydrogen hubs,” that is, colocated self‐sustaining regional systems of hydrogen production and use.

A national vision for a hydrogen economy was first published in the United States as early as 2002, when a consortium of 53 senior executives from industry, universities, environmental groups, federal and state government agencies, and National Laboratories came together to discuss hydrogen development in the United States. The subsequent report put out at that time highlighted the need for federal and state‐level energy policies that encourage hydrogen, as well as “strong public–private partnerships” (U.S. Department of Energy, 2002). The newest National Hydrogen Roadmap was published in June 2023 (Satyapal et al., 2023).

The United States currently produces about 10 million metric tons of hydrogen per year (U.S. Department of Energy, 2021). The National Clean Hydrogen Strategy and Roadmap targets the production of 50 million metric tons of hydrogen per year by 2050 (Satyapal et al., 2023). Today, fossil fuel feedstocks account for 99% of hydrogen production, with 95% from natural gas using steam methane reforming (SMR) without carbon capture and sequestration (CCS) (U.S. Department of Energy, 2020a). Most of this hydrogen is used in oil refineries and in the production of ammonia, 80% of which today is used in fertilizer production. Globally, only 0.7% of hydrogen is produced via electrolysis or SMR with CCS (International Energy Agency, 2019b).

In this review, we focus on the two most widely recognized pathways for producing hydrogen without CO_2_ emissions. These are (1) steam reforming of methane (SMR) with CCS and (2) the use of an electrolyzer powered by near‐zero GHG emissions electricity to split water (H_2_O).

The SMR pathway produces roughly 5.5 kg CO_2_ for every kg of hydrogen produced. This value would be higher if the efficiency is lower than the assumed value of 65% (Hauglustaine et al., 2022; Komarov et al., 2021). Although there is ongoing work on electrolyzer efficiencies (Hodges et al., 2022; Su et al., 2024), electrolysis of water currently requires approximately twice as much energy (in the form of electricity) to produce 1 kg of hydrogen compared with the SMR process. Electricity, which must in turn be generated from other primary energy sources with associated efficiency losses, accounts for an overwhelming share of the cost of producing hydrogen via this pathway. Table 1 briefly summarizes the two pathways.

**TABLE 1:** Overview of the two hydrogen production pathways considered in this analysis. A separation efficiency of 65% is assumed for SMR and of 70% for electrolysis using proton‐exchange membrane (PEM) fuel cells.

Pathway	Steam methane reforming	Electrolysis
Chemical reaction(s)	CH_4_(*g*) + H_2_O(*g*) (+heat) ⇄ CO(*g*) + 3H_2_(*g*) CO(*g*) + H_2_O(*g*) ⇄ CO_2_(*g*) + H_2_(*g*) (+small amount of heat)	2 H_2_O(*l*) → 2 H_2_(*g*) + O_2_(*g*)
Share of U.S. hydrogen production in 2020 (U.S. Department of Energy, 2020c)	95%	1%
Feedstocks and energy required for 1 kg hydrogen	Reactant(s): 3.1 kg methane, 6.9 kg water Energy: 7.8 kWh Coproducts: 5.5 kg CO_2_	Reactant(s): 9.0 kg water Energy: 15.4 kWh Coproducts: 8.0 kg O_2_
To be low carbon, this pathway requires…	Installation of CCS and continuous high CO_2_ removal rates during operation	Use of zero‐carbon electricity

Although there is an urgent need to transform the energy system, there are also risks inherent in any such large‐scale system change. In the United States, the Department of Energy (DOE) is supporting regional clean hydrogen hubs that will concentrate regional production and use. These hubs are envisioned as forming “the foundation of a national clean energy network” (Hodges et al., 2022; Su et al., 2024). Arguments for focusing on regional hubs include possible greater ease of coordination among buyers and suppliers and the potential to avoid relying on long‐distance transport networks. However, adopting a hubs approach may do little to mitigate, and may even magnify, other risks. Our goal in this review is to describe the major sources of risk that may arise in hub development, provide an integrated characterization of those risks, and examine the extent to which those risks can be mitigated through careful design. We consider two categories of risk: (1) economic risk that projects will result in stranded assets and (2) environmental risk that projects will not contribute to reducing, and could even exacerbate, GHG emissions. We take a systematic approach to characterizing the state of knowledge surrounding both categories of risk. We conclude by outlining strategies for mitigating the risks we identify.

## 2 METHODOLOGY

In support of this review, we performed a keyword search of the peer‐reviewed literature since 2000 and augmented it with a Google search of the gray literature, focusing on major reports that are published annually and reports by U.S. National Laboratories. Among the articles identified through our initial searches, we then focused on those related to the production and use of hydrogen as a fuel or energy carrier.

As shown in Table 2, the resulting discussion is divided into two broad categories: “economic and social risks” and “environmental risks.” For economic and social risks, we first chronicle estimates of the cost of producing hydrogen using different methods and then discuss factors that could further affect cost or other drivers of investment, including technical performance (e.g., materials embrittlement, availability of carbon capture and storage), contracting for offtake, workforce implications, policy delays, and public opposition. For environmental risks, we focus on hydrogen leakage, methane leakage (in the case of processes that use natural gas as a feedstock), and local air pollutant emissions.

**TABLE 2:** Sources of risk discussed in this review.

Economic and social risks	Environmental risks
Production cost	CO_2_ emissions not displaced
Technical performance	Hydrogen leakage
End‐use demand	Fugitive methane emissions
Workforce implications	Local air pollution
Public opposition	

## 3 ECONOMIC AND SOCIAL RISKS

### 3.1 Risk that production costs will be higher than anticipated

**FIGURE 1:**
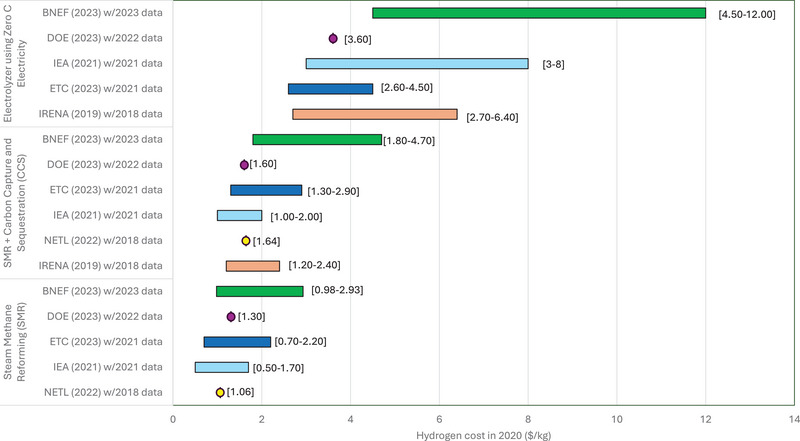
Published estimates of the levelized cost of hydrogen production using electrolysis of water (top), SMR with CCS (middle), and SMR without CCS (bottom). Dates reported in parentheses are for the year in which the reports were published (BloombergNEF, 2023; ETC, 2021; International Energy Agency, 2021; IRENA, 2019; E. Lewis et al., 2022; U.S. Department of Energy, 2023d).

The cost of production will drive the affordability of hydrogen as a fuel, as a medium for long‐term energy storage, and as an input to industrial processes and other end uses. If project or operating costs are higher than expected, investor returns will be lower than expected and may slow or end project or system development. Figure 1 reports cost estimates for hydrogen produced via SMR with and without CCS as well as via electrolysis using decarbonized electricity.

Cost estimates for hydrogen produced through SMR, both with and without CCS, are smaller today compared with the decarbonized electrolysis route. When CCS is used, this hydrogen is sometimes termed “blue” hydrogen. Although electrolysis is currently expensive, anticipated reductions in the cost of both electrolyzers and delivered renewable electricity could reduce the cost per kilogram to around U.S. $2/kg over the next decade (Lazard,^2021^). Hydrogen produced via electrolysis using renewable energy is sometimes called “green” hydrogen.

The cost drivers of the two production routes are very different. Today, it is possible to purchase electrolyzers “off‐the‐shelf” as package units. However, it is our understanding that to date steam reforming systems have all been custom engineered. If package units for steam reforming become available, costs for hydrogen made via SMR might be lower. Compared with systems manufactured in Europe and the United States, capital costs for electrolyzers made in China are estimated to be four times lower ($300/kW versus $1,200/kW), although, at 60,000 hours, operating lifetimes are currently estimated to be shorter than the 80,000 hours achieved by some systems (Heyward, 2022). As in the case of solar PV systems, the production of electrolyzers in China is heavily subsidized at the national and provincial levels (Heyward, 2022).

The DOE and National Energy Technology Laboratory (NETL) point estimates are their assessments of the average values for the United States. These values will vary depending on production volumes and the local costs of electricity and natural gas. The ranges shown for IRENA, ETC, BloombergNEF, and IEA use data from a number of countries. The wide range of costs estimated for electrolysis from BloombergNEF arise because the data included higher costs of manufacturing from certain countries in Southeast Asia (BloombergNEF, 2023).

The estimates for the cost of SMR with CCS include several different assumptions about the rate of carbon capture. Much of the cost range for electrolysis reflects the source of renewable energy (solar, wind). Although the upper bounds on cost estimates for hydrogen produced via electrolysis are generally higher than those for SMR, most literature suggests that as renewables prices and the cost of electrolyzers continue to fall, hydrogen produced via electrolysis may become the lower cost option (BloombergNEF, 2023; ETC, 2021; IRENA, 2019; Lazard, 2021). Some argue that the cost of hydrogen made through electrolysis could be comparable to or lower than alternative production methods as early as 2030 (BloombergNEF, 2023; ETC, 2021). Finally, policies that place direct or indirect costs on releasing CO_2_ into the atmosphere will also shape comparative costs.

Scale is an important driver of production costs in industries where fixed costs are high relative to variable costs. However, starting with larger volumes raises the risk that not all the hydrogen produced will be sold. Using the NREL H2A Modeling Suite (NREL, 2018), in Table 3 we compare the unit production costs for “small” and “large” designs (defined by the allowable range of capacities in the H2A model, indicated in parentheses in the “Scale” column) for both centralized and distributed SMR and electrolysis pathways. Centralized production involves a much larger average range of production compared with distributed production and may involve different technology configurations. We further compared estimates for current and future systems. In both cases, we examinesensitivity to the cost of the input that comprises the largest driver of unit cost—the cost of natural gas for the SMR and the cost of electricity for the electrolysis pathway.

**TABLE 3:** Sensitivity of hydrogen production costs to assumptions about scale, time frame, and feedstock or electricity input price, by process. PEM is proton‐exchange membrane; SO is solid oxide.

			Current		Future	
Process	Scale		“Small”	“Large”	“Small”	“Large”
SMR with CCS	Centralized (235–425 t/day)		$1.20	$1.14	$1.36	$1.31
	Distributed (500–6,000 kg/day)		$1.74	$1.36	$1.78	$1.52
	Central (235–425 t) with high CH_4_ price ($9/mmBtu)		$5.97	$5.91	$5.91	$5.87
Electrolysis	Centralized	PEM (20–200 t/day)	$5.88	$4.42	$5.13	$4.31
		SO (30–70 t/day)	$4.71	$5.04	$3.81	$3.78
	Distributed	PEM (500–6,000 kg/day)	$5.33	$4.79	$4.81	$4.49
	Central with high electricity price ($0.14/kWh)	PEM (20–200 t/day)	$9.70	$8.24	$8.38	$7.56

Overall, SMR costs are lower today, although depending on the relative prices of natural gas and electricity, the cost of producing hydrogen via electrolysis compared with the SMR route when the natural gas cost is high ($9/mmBtu) is roughly equal. Doubling the assumed electricity price increases the cost of production from electrolysis (in this case, for PEM) by 1.8 to 8 times, depending on the cost of natural gas. Economies of scale are projected to be largest for all electrolysis systems and for distributed SMR systems, but centralized SMR systems show limited latitude for cost reduction. Comparing current and future projections of the cost of hydrogen production from electrolysis, potential cost reductions are expected to be larger than for the SMR process, although they do not drop as low as estimates identified by some studies (see Figure 1).

### 3.2 Risk of technical performance failures or infrastructure gaps

Three types of infrastructure will be critical to support the future production and use of hydrogen at scale: (1) pipeline networks for hydrogen transport and distribution; (2) CCS including approved sequestration sites as well as pipelines or other means of transporting CO_2_; (3) electric power transmission infrastructure to supply electricity for electrolysis and to deliver electricity if it is made from hydrogen.

#### 3.2.1 Hydrogen transport infrastructure

Although there are several methods for transporting hydrogen, with varying price points and associated challenges and risks (Caliendo & Genovese, 2021; U.S. Department of Energy, 2022b, 2022c), the most efficient for large volumes is via pipeline (International Energy Agency, 2019a). One important risk involves maintaining hydrogen pipeline integrity against the risk of cracking or failure. At temperatures below about 150°C, hydrogen molecules diffuse into iron and steel, leading to embrittlement and cracking of conventional carbon steel pipelines (Dwivedi & Vishwakarma, 2018). Hydrogen embrittlement could result in major leaks, not just from pipelines but also from a variety of other metallic fixtures in gas systems (Hafsi et al., 2018; Somerday & San Marchi, 2006).

Existing natural gas transmission infrastructure can transport up to 5%–15% hydrogen by volume without encountering serious problems with embrittlement (Melaina et al., 2013). In the context of a hydrogen hub, risks that scale with the length of transport infrastructure required will be initially limited, as transportation of hydrogen is likely to rely on shorter lengths of new dedicated pipelines. However, if hydrogen production is scaled up and perhaps some is blended into the existing transport infrastructure of various vintages and technical specifications, a new set of challenges will emerge. Moreover, if hydrogen becomes a commodity, cyclic loading and fluctuations in pressures and volumes are likely to occur through the transportation and storage stages—both of which contribute to embrittlement (Somerday & San Marchi, 2006) and to fatigue of steel pipelines (Bouledroua et al., 2020; Melaina et al., 2013). Melaina et al. (2013) reports that hydrogen embrittlement of steel pipes used in high‐pressure natural gas transmission lines is likely to be greater than for low‐pressure distribution lines—a risk that may be easier to manage in hydrogen hubs if shorter transport distances translate into a more limited need for high‐pressure transmission and the construction of new hydrogen‐compatible pipelines. Additionally, there are options for pipeline modifications to better withstand hydrogen embrittlement, including coatings (Bhadeshia, 2016) as well as the chemical composition of the steel used (Cai et al., 2022).

If the existing natural gas transmission infrastructure is repurposed, the operating temperature (Xu et al., 2024), the pipeline age (Nykyforchyn et al., 2021), the blending ratio, and the pipeline steel material strength (Zhang et al., 2023) are key factors in determining susceptibility to corrosion and embrittlement. Within the United States, there are limited numbers of pilot projects that have tested hydrogen blending (with the highest being in Hawaii with a 12% blend by volume; Topolski et al., 2022), leaving room for future pilot projects to further explore higher blends as hub project specifications will emerge for transmission and end‐use applications. In the context of a hydrogen hub, risks that scale with the length of transport infrastructure required will be initially limited, as transportation of hydrogen is likely to rely on shorter lengths of new dedicated pipelines. However, if hydrogen use is scaled up via blending into existing transport infrastructure of various vintages and technical specifications that vary in their capability to accept hydrogen, a new set of challenges will emerge.

**FIGURE 2:**
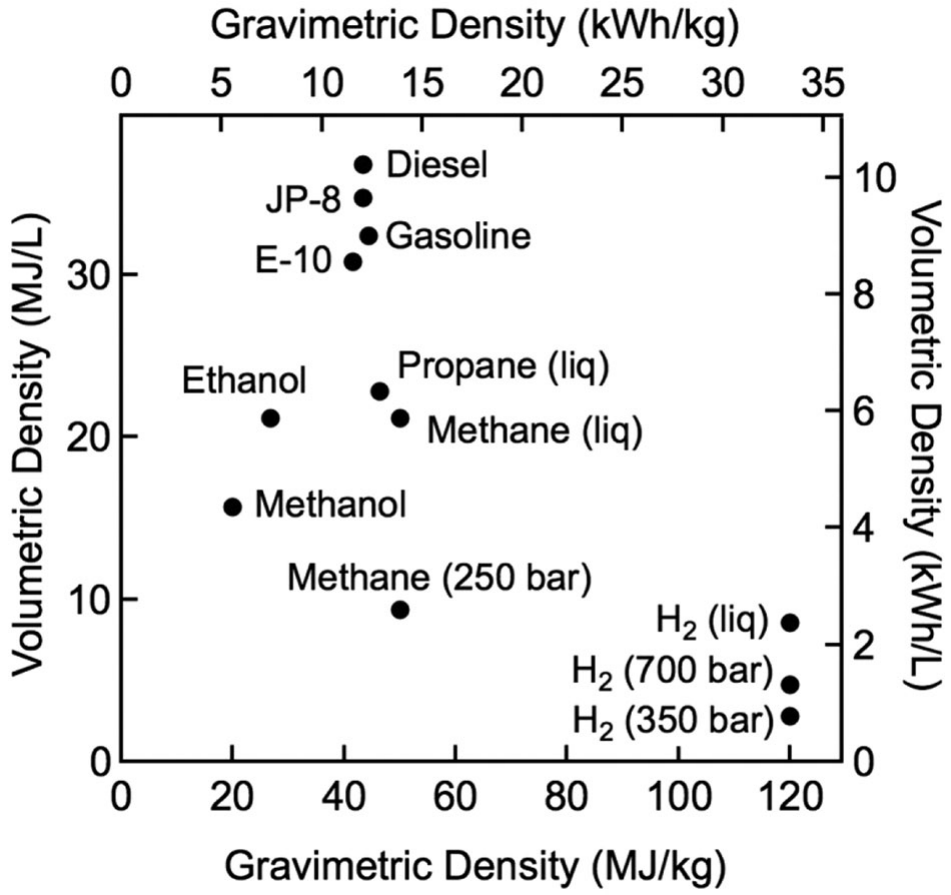
Although hydrogen has a high energy density (in terms of MJ/kg), the required storage volume is much larger than for more conventional fuels (U.S. Department of Energy, 2020b). This complicates applications in which volume is critical such as on aircraft.

Pipelines for pure hydrogen require specialty steels or nonmetal pipes, such as fiberglass‐reinforced pipe or high‐density polyethylene pipe (HDPE) (U.S. Department of Energy, 2018). Existing natural gas pipelines can often be replaced using minimally invasive trenchless pipe installation or upgraded with interior coatings (NASTT, 2022). Such a strategy can simultaneously support new development without stranding existing assets. New hydrogen transmission infrastructure, which might be installed alongside existing pipeline ROWs, will be needed for the successful implementation of hydrogen fuel networks. Because of its lower density (Figure 2), moving hydrogen requires more energy than moving natural gas. This will not be a serious issue for an early hub that uses the hydrogen it produces locally, but it could become an important consideration as a hub begins to move hydrogen over long distances.

As with natural gas, hydrogen can be stored as a compressed gas in tanks or geologic formations. However, hydrogen's low density presents a variety of challenges for storage, including the need for larger volumes than those involved when storing natural gas, larger amounts of energy to perform compression, and higher potential rates of leakage. Like natural gas, hydrogen can also be liquified for more compact storage, although liquid hydrogen still requires more storage volume than LNG for an equivalent energy content (Figure 2). Higher storage density (MJ/kg) can also be achieved through conversion to a denser compound such as ammonia (Andersson & Grönkvist, 2019). In most early hubs, storage of hydrogen will probably take the form of compressed gas in tanks. Although there is commercial‐scale hydrogen storage in salt caverns like those being used for natural gas, and large‐volume geologic storage could provide economic advantages (Moran et al., 2023), potentially high leakage rates from other geologic formations will require careful assessment and could undermine project economics. Recently, it was announced that Aces Delta, a joint venture of Mitsubishi Power Americas and Magnum Development LLC, will build an underground hydrogen storage facility in Delta, Utah, with a capacity of 300 GWh, with funding support from the U.S. Department of Energy (Bellini, 2022).

Siting of pipelines for hydrogen must undergo regulatory review and may be subject to delays or public resistance. Parfomak (2021) provides a comprehensive review with extensive references on the regulatory, research, and policy issues related to the pipeline transportation of hydrogen.

#### 3.2.2 Availability of CCS

Producing hydrogen from natural gas without adding additional CO_2_ to the atmosphere will require carbon capture and the use or disposal of the resulting CO_2_. Although recent years have witnessed growing discussion of “carbon utilization,” many of those uses tie up CO_2_ only briefly before it enters the atmosphere, only partially displacing CO_2_ emissions, or in the case of using captured CO_2_ for enhanced oil recovery can end up resulting in net positive emissions.

Multiple risks arise from the technical performance of carbon capture systems (Collodi et al., 2017; Oni et al., 2022; Ruether et al., 2005; Sadler et al., 2016). Many involve nontechnical issues, including a lack of effective carbon pricing and predictable energy policy, clear and consistent CCS regulation, and public acceptance (Bui et al., 2018; de Coninck & Benson, 2014; Herzog, 2011; Lau et al., 2021; Lupion et al., 2015; M. G. Morgan & McCoy, 2012). In Europe and Asia, CO_2_ movement between countries is governed by the London Protocol and Basel Convention, but not all nations are signatories to these, meaning current international law is insufficient to manage the passage of CO_2_ across national boundaries, including its subsurface injection and disposal (Lau et al., 2021). Although many countries have some form of carbon pricing policy implemented or under development (International Carbon Action Partnership, 2023; The World Bank, 2023), the carbon price is generally too low to spur private investment in carbon emissions reduction on its own. Early adopters of hydrogen hubs will likely rely more heavily on specific hydrogen production incentives (Kneebone, 2023; Majid, 2023). Oni et al.’s (2022) techno‐economic assessment of carbon pricing on the LCOH for hydrogen production via SMR would only become the less expensive option compared with a system that includes CCS at a carbon price of $125/ton (Oni et al., 2022).

In the United States, the recently increased value and eligibility for the 45Q tax credit has boosted CCS development, but progress is still slow. In the United States, the Pipeline and Hazardous Materials Safety Administration (PHMSA) within the Department of Transportation holds both safety and regulatory authority over the pipeline transportation of CO_2_ in a supercritical state (Daugherty, 2023). In light of the CO_2_ pipeline failure that happened in 2020 in Satartia, Mississippi, PHMSA has initiated a new rulemaking that will amend their Pipeline Safety Regulations (49 CFR 190–199). The notice of proposed rulemaking was published in April 2024 (U.S. Department of Transportation, 2024). The issues of pore space ownership and property rights are still undetermined in most U.S. states, and there is no federal guidance related to pore space or the long‐term liability of sequestration sites (de Coninck & Benson, 2014; Lupion et al., 2015; M. G. Morgan & McCoy, 2012).

In many nations, property rights for use of the subsurface are held at the national level, but this is not the case in the United States. Efforts a decade ago to develop a national framework to address the issues of subsurface property rights, injection field amalgamation, licensing of injection sites, long‐term monitoring, and liability were unsuccessful (M. G. Morgan & McCoy, 2012). The EPA has developed a permitting framework under the Underground Injection Control (UIC) program for all classes of injection wells (with Class VI applying to the geologic sequestration of CO_2_) that is rather narrowly focused on the protection of underground sources of drinking water (U.S. Environmental Protection Agency, 2023) and does not adequately address these other key issues.

Before a developer can be granted a Class VI permit, they must perform a detailed characterization of the subsurface geology and secure approvals from surface property and/or mineral rights owners. Although some states have been developing formal arrangements to facilitate reservoir consolidation and deal with situations in which a minority of property owners are unwilling to grant approval, the development of such processes is still in a very early stage in most U.S. states.

There is a risk of delays in the regulatory processes required to advance CCS. A Class VI permit application for an injection well into a characterized reservoir must be submitted to the appropriate EPA Regional Office (U.S. Environmental Protection Agency, 2024). Once a developer submits their application package to the EPA's online portal, the permit review process begins. The EPA estimates it will take 18 to 24 months (Harvey & James, 2021) to complete this process and approve a permit application. This estimate assumes that the process goes smoothly and any appeals filed do not impact the project timeline. This estimate also does not include the additional time required for preparing an application and obtaining authorization to inject. An approved Class VI permit only gives the developer permission to construct the injection well and collect preoperational data. The developer then must submit the test data and other information back to the EPA and wait to receive authorization to inject. Depending on the magnitude of differences in the observed versus expected geology, the timeframe to receive authorization to inject is highly uncertain, and there is the possibility that a permit modification could be required. If a significant permit modification is necessary, the applicant must update their application package and go back through the full review process, including the public comment period and an additional opportunity for appeals to be filed (EPA Staff, personal communication, 2022). This means that the period between receiving a permit to drill and authorization to inject involves additional time and a substantial degree of uncertainty. Since promulgating the UIC Class VI rule in 2010, the EPA has approved only six Class VI permits.

Moore et al. (2023) used expert elicitation combined with stochastic simulation to estimate the total time required to develop an injection well and secure approval, focusing on Southwestern Pennsylvania. They concluded that the development process may take “at least 4 years, and perhaps more than 12 years.” The Biden administration's public communications recognize that such a long delay can seriously impede the development, not just of hydrogen hubs, but of many other applications of CCS that are likely to play an important role in decarbonizing the energy system (The White House, 2023). The administration is looking for ways to speed the process, but a variety of legal and regulatory barriers remain.

Under the UIC program, the EPA can delegate primary enforcement authority, or “primacy,” to a state for specified well class permitting upon approval of a state primacy application. It is widely believed that state primacy for Class VI wells will accelerate the permitting process, although some in the environmental community have expressed concerns that this could result in a less careful review. Currently, only North Dakota, Wyoming, and Louisiana have obtained Class VI primacy. North Dakota applied for primacy in 2013 and received it in 2018 (Harvey & James, 2021). Wyoming started working closely with EPA Region 8 in 2008, when their legislature developed statutes to regulate CCS in the state and began discussing Class VI primacy with the EPA in 2016 (Barkau, personal communication, February 28, 2022). In January 2018, Wyoming submitted its primacy application package to the EPA, and primacy was granted in October 2020 (Barkau, personal communication, February 28, 2022; Harvey & James, 2021). In April 2021, the state of Louisiana Department of Natural Resources, Office of Conservation, Injection, and Mining Division submitted a Class VI primacy application to EPA Region 6 (Louisiana Department of Natural Resources, 2021); Louisiana was granted primacy (U.S. Environmental Protection Agency, 2024).

Motivated largely by the growing interest in the production of hydrogen from natural gas with CCS, a number of states have recently begun the application process or are in various stages of working toward developing such applications. Arizona, Texas, and West Virginia are at various stages in the process of applying for Class VI state primacy (U.S. Department of Energy & U.S. Environmental Protection Agency, 2022).

As noted above, the current EPA regulations, and presumably the criteria that will also be used by states, are motivated by concerns about groundwater protection. Despite early calls to consider long‐term stewardship, liability, and similar issues at the national level, they have yet to be systematically addressed (M. G. Morgan & McCoy, 2012). Inadequate attention to these issues in the short‐term could give rise to serious long‐term complications.

#### 3.2.3 The need for electric power transmission capacity

Producing hydrogen from water via electrolysis requires electricity. Securing electricity supply for use in early U.S. hubs is unlikely to be a serious problem, although it may be difficult to meet U.S. Treasury requirements to qualify as sourcing decarbonized electricity, which include time matching and other provisions. However, as the use of electrolysis expands, total supply of electricity could become a serious bottleneck. After years of stable demand, U.S. electricity demand is now beginning to grow as a result of the proliferation of datacenters and other computing applications such as artificial intelligence and crypto currency. The electrification of transport and industrial processes is also beginning to make significant contributions to demand growth (Halper, 2024; Plumer & Popovich, 2024). Future supply is likely to be further constrained by the lack of transmission capacity. In its 2023 *National Transmission Needs Study* (U.S. Department of Energy, 2023c), the U.S. Department of Energy estimates that if the economy remains healthy and the country wants to meet its decarbonization goals, regional and interregional transmission capacity will need to more than double over the coming decades. For many decades it has been difficult, and sometimes impossible, to build new overhead high‐voltage AC transmission lines in the United States (Vajjhala & Fischbeck, 2007). However, some strategies can be employed to increase substantially the amount of power moving through existing transmission corridors (Reed et al., 2020). In addition, newer technology for high‐voltage DC cables can allow the use of transmission through nontraditional rights of way.

### 3.3 Economics of future end uses and offtake agreements

Any successful hydrogen economy will require both a steady supply and reliable demand for the product. Today, hydrogen demand in the United States is primarily concentrated along the Gulf Coast where it is used in petroleum refining. In interviews conducted with experts involved in the development of hydrogen hubs, some have expressed concerns about whether demand will grow sufficiently to sustain a hydrogen market (Iacob & Morgan, 2024). Several strategies could be pursued to encourage demand growth: (1) the federal government could promote clean hydrogen demand through contracting requirements for federal facilities such as Department of Energy labs, military bases, etc. (Bajema et al., 2023); (2) state and/or federal offices could support research on novel applications of hydrogen use, including the encouragement of cooperation across academia, national labs, and industry; (3) vertical integration within companies (or several companies or other end users through joint ventures) could allow more internal control over several areas of the hydrogen value chain (Sandstrom et al., 2022); (4) regulatory and permitting changes could be used to encourage midstream delivery options for wide geographic areas to encompass larger set of potential offtakers; (5) development of standards across hydrogen production could ensure competitive and systematic labeling of products; (6) education for government and business stakeholders could lead to better understanding of the nuances and opportunities for integrating hydrogen (Iacob & Morgan, 2024); and (7) similar to the 45V tax credits for producers, financial incentives could be included for hydrogen offtakers.

The economics of hydrogen relative to other fuels or energy carriers should be a key factor in determining appropriate early uses of hydrogen. Generating electricity, for instance, is unlikely to be a good first‐use case. Because all the strategies to produce hydrogen require energy, there is a large efficiency penalty associated with using hydrogen to generate baseload electricity, instead of using the input energy (e.g., natural gas) directly. Even if the electricity comes from a carbon‐free source, it would be more efficient to deliver it directly to the grid. The primary advantage of hydrogen in the context of electricity is the ability to store energy for later use (EPRI, 2023). If the technology did not already exist, direct use might also be justified on the basis of demonstrating gas turbine designs that can use 100% hydrogen. However, in as much as such turbines do exist (GE, 2022), there may not be an advantage to conducting such a demonstration.

In contrast, if the source of the energy used to produce the hydrogen is wind or solar, and a gas turbine using stored hydrogen is used to generate electricity when those intermittent sources are unavailable, the demonstration value of such a system may be greater. Such a demonstration would be most compelling if the generation plant were colocated with production and storage facilities at the hub (see discussion below of hydrogen transport and storage) (EPRI, 2023). However, such a system would still need to compete with other energy storage or demand response approaches.

Hubs might also be colocated at industrial sites where hydrogen can be used to decarbonize otherwise carbon‐intensive industries. Although some of the CO_2_ produced in cement making comes from the feedstocks, hydrogen can be used as a drop in fuel to replace natural gas, reducing CO_2_ emissions intensity (Norster, 2023). Steel plants could also become major users of hydrogen. Production of direct reduced iron (DRI) for steelmaking is an example of one of several applications in heavy industry that could use hydrogen to lower CO_2_ emissions. A good example of this is the H2Stahl project in Duisburg, Germany, in which ThyssenKrupp, Air Liquide Deutschland, and VDEh Betriebsforschungsinstitut (BFI) are collaborating to replace blast furnaces with ironmaking in direct reduction reactors (ThyssenKrupp, 2022). The development of DRI is still in a very early stage in the United States, but recently the DOE awarded funding to the Cleveland‐Cliffs Middletown facility to undertake a similar retrofit. Given that such systems are yet to be built, and, in contrast to other early‐stage applications, would likely require fairly high production volumes, they should be located in places with abundant resources for producing hydrogen. Developers should also remain acutely aware of alternatives, such as molten oxide electrolysis, which may prove to be more cost effective if sufficiently inexpensive and abundant sources of clean (near‐zero CO_2_ emissions) sources of electricity are available (Stinn & Allanore, 2020).

Fueling sites for fuel cell vehicles could also support demand, both at a hub and at other locations. Today, in North America, there are more than 50 such stations in California and five in Canada (U.S. Department of Energy, 2024a). However, the growth of new stations has been slow outside of a few major metropolitan locations. In some places, planners and developers have focused on heavy‐duty and high‐use commercial vehicle fleets and locomotives (Day, 2022), ships (Gallucci, 2021), and aviation (Dray et al., 2022). Many states have active programs to subsidize the adoption of new decarbonized transportation fuels. To date, those state programs have largely subsidized bio‐based fuels, but the enabling legislation could typically also support hydrogen fueling (IRS, 2024; Maryland EV, 2023).

There may be a need to refine and streamline pipeline regulation, in terms of both siting and the jurisdiction of rates for interstate blended natural gas. Hydrogen pipelines may be classified as common carriers—“a legal classification which requires them to serve all shippers at all times and typically makes their rates subject to economic regulation through regulated tariffs” (Parfomak, 2021).

Lastly, some end users may impose contract requirements for the CO_2_ emissions intensity of hydrogen production, as Europe has indicated it may do for its imported hydrogen in the future (Erbach & Svensson, 2023). Contractual demands pose an additional caveat for producers to consider. They also raise the importance of developing common or interoperable frameworks for measuring CO_2_ emissions intensity of production consistently.

### 3.4 Risk of inadequately trained or unavailable workforce

Hydrogen hubs are expected to create a range of new jobs (Bezdek, 2019), but the composition of jobs and time horizon will vary, depending on hydrogen production technology and end uses. Building hubs will create jobs in construction, likely in the near term. Operating hubs that use natural gas as a feedstock may preserve jobs in the oil and gas sector and expand jobs adjacent to refining and chemical production. It is unclear whether these jobs will transfer directly from other at‐risk industries, including potentially fossil fuel extraction and uses that are hard to abate or substitute, or whether they will expand employment, for example, by attracting workers from outside of the hub region.

Projecting occupational matches for workers using spatially resolved data on skills, knowledge, abilities, salary, and other relevant characteristics could help to assess employment prospects that accompany a hydrogen hub or other large energy and infrastructure investment (Miles et al., 2023). These approaches can also help to identify training gaps and complement macroeconomic modeling exercises that project aggregated impacts on a region's economic structure and workforce (Mayfield et al., 2023). In assessing the impacts of transition, attention should be paid to changes in employment along the entire supply chain. For example, a shift to direct reduced iron in steelmaking may ultimately render coke works obsolete, eliminating a major employer associated with many integrated iron and steel mills. The enablers of successful occupational transitions in practice are the subject of much speculation and limited research, suggesting an important direction for future work.

### 3.5 Risk of public opposition

Public opposition to hydrogen can be expected to take different forms, depending on the feedstock from which it is produced. In the case of hydrogen produced from natural gas, multiple steps involve potential targets of public opposition, in particular, pipeline siting and CCS infrastructure siting and operations, as well as more general opposition to hydraulic fracturing and any continued use of fossil fuel, even if CO_2_ emissions are captured. In the case of hydrogen produced from renewable electricity, opposition is more likely to focus on the buildout of wind and solar, to the extent that the public objects for aesthetic or wildlife conservation reasons. Here, we focus primarily on the risk of public opposition to CCS and hydrogen.

#### 3.5.1 Opposition to CCS

Almost every time a CCS project has been proposed, it has met with public opposition based on a variety of concerns (Pianta et al., 2021; Whitmarsh et al., 2019). Two decades ago, Morgan and Bruine de Bruin undertook some of the first studies of issues of public perception and acceptance of CCS technologies (Palmgren et al., 2005). More recent studies have continued to show relatively modest levels of public understanding and substantial levels of concern, which could easily evolve into serious opposition when CCS is discussed in isolation. Some research also suggests a greater potential for acceptance if the use of CCS can be framed comparatively in terms of alternative technologies and the broader challenge of decarbonizing the energy system (Braun, 2017; Broecks et al., 2021; Fleishman et al., 2010; Krause et al., 2013; Tcvetkov et al., 2019; Witte, 2021).

A consistent finding across more than a decade of studies that have examined public perceptions of CCS is that most U.S. respondents have not heard of the technology. This continued to be the case as recently as 2018 in a study of a demographically representative sample of 1,520 U.S. residents (Pianta et al., 2021) as well as in a more recent study by Iacob and Morgan (Iacob & Morgan, 2023). Several studies have suggested that how the issues around CCS are initially framed and what role, if any, communities have in decision‐making, can have a big impact on subsequent public assessments. Case studies more than a decade ago that examined two communities in California that were potential sites for experimental sequestration projects found that “communities were concerned that inadequate knowledge of carbon sequestration could lead to mistakes during the injection of CO_2_” (Wong‐Parodi & Ray, 2009). Early studies at Carnegie Mellon University found similar results—including a desire by participants to understand available alternatives. In fact, in a 2005 study, it was suggested that the public may not support CCS because it could be a temporary solution that could “[create] future problems” (Palmgren et al., 2005). An attempt to develop a CCS pilot project in Germany encountered sufficiently strong public opposition that it resulted in the termination of the program (Slavin & Jha, 2009).

There are a number of operating CCS projects, including two large ones off the coast of Norway associated with offshore natural gas production. Norway has also launched a project called “Longship,” which will provide a sequestration capability for a variety of commercial sources of CO_2_ (CCS Norway, 2023). After Germany abandoned an early effort to demonstrate geologic storage because of public opposition, roughly a decade later in 2019 Chancellor Angela Merkel announced that the country would put CCS back on the table as an option (Wettengel, 2020). In the United States, most projects that have involved injections of substantial volumes of CO_2_ are associated with enhanced oil recovery, a process that produces net positive carbon footprints after the first few years of production. Despite a variety of early undertakings, such as the FutureGen project, the United States has yet to complete any large program of CCS motivated strictly by reducing CO_2_ emissions to the atmosphere. The Department of Energy has begun several programs since recent legislation (specifically, the Bipartisan Infrastructure Law and the Inflation Reduction Act) was passed, which hope to see significant advancement in the CCS industry (Fahs et al., 2023).

In addition to a variety of general concerns about safety, and a belief by some that trying to dispose of pollutants is somehow wrong (Tarr, 1996), induced seismicity (USGS, 2022) has also emerged as a concerning issue. Two notable examples, neither of which is directly linked to CCS but clearly indicative of possible problems, include the termination of a geothermal project in Switzerland (Glanz, 2009) and recurring issues that arose in Oklahoma as a result of wastewater injection (Chokshi & Fountain, 2016; Hincks et al., 2018). The risk of induced seismicity can be managed with an appropriate choice of injection site. In some cases, it may require the extraction and surface treatment of the brine that will be displaced when CO_2_ is injected. This could become quite expensive and present environmental and other problems.

Although the Class VI process is designed to prevent this, there are also concerns that CO_2_ could leak from its subsurface reservoir and reach drinking water aquifers. If this were to happen, the CO_2_ would acidify the water, which would potentially make heavy metals more soluble and increase their amounts in drinking water (Apps et al., 2010).

#### 3.5.2 Opposition to using hydrogen

In addition to the issue of public perception of CCS discussed above, more general public perceptions of hydrogen and its use are likely to have a profound effect on the acceptance of hydrogen hubs and more generally a hydrogen economy.

There has been limited study of public perception of hydrogen in the United States (Schmoyer et al., 2006); most studies have been conducted in Europe and Asia (Emodi et al., 2021). These studies have mainly focused on hydrogen fuel cell vehicles and hydrogen fueling stations, and most questionnaires and surveys have been done in regions where the participants had some exposure to hydrogen through a previously deployed project or an on‐site demonstration prior to the survey (Alanne, 2018; Bellaby & Clark, 2014; Hienuki et al., 2019; Itaoka et al., 2017; Trencher, 2020). Findings suggest that the public is generally supportive, although lacks knowledge of the topic. Discussion‐format studies have found that the general public places importance on ongoing conversations between the public and the developers (Ashworth et al., 2019), the importance of safety and risk management (Bellaby & Clark, 2014; Markert et al., 2007; Ono & Tsunemi, 2017), and the emotionally‐driven responses participants have when presented with a new hydrogen project (Huijts, 2018).

Findings from the recent Iacob and Morgan (2023) study of public understanding of hydrogen in Southwestern Pennsylvania were similar to many of those in the literature, including a low level of familiarity with hydrogen (Iacob & Morgan, 2023). However, participants displayed unique regional considerations, by discussing historical industrial impacts, potential local environmental benefits, and the engagement of local skillsets associated with local natural gas production. Given the present state of the literature, it is impossible to judge the likelihood that a hydrogen hub that involves natural gas and the use of CCS will encounter opposition. As reported in Iacob and Morgan (2023), some may have a strong aversion to any continued use of fossil fuel, and some may have a similar aversion to the use of deep geologic sequestration of CO_2_—especially if the injection projects are developed in their geographic vicinity (Iacob & Morgan, 2023). Others who see the use of natural gas and the development of CCS as a source of continued regional economic activity may find such developments beneficial.

It will also be difficult to predict the public response to hydrogen produced by electrolysis, using zero‐carbon electricity sources. As recently as last year, an experimental project proposal to blend hydrogen produced via electrolysis into natural gas was scrapped in Eugene, Oregon, after concerns regarding health, safety, and social justice were raised by the community stakeholders (Baumhardt, 2022). Studies suggested that public opposition to hydrogen projects could be mitigated by repeated, timely discussions between project developers, respected subject‐matter experts, and stakeholders; it is imperative that such discussions provide ample time for stakeholder feedback to be integrated into the project design (Ashworth et al., 2019).

## 4 ENVIRONMENTAL RISKS

Although hydrogen is a carbon‐free energy carrier, its use still poses several important risks to the local and global environment.

### 4.1 CO_2_ emissions not displaced

There are a number of reasons why in practice energy‐ and process‐related CO_2_ emissions reductions associated with a shift to using hydrogen over alternative feedstocks may not result in the magnitude or even direction of GHG mitigation progress expected. These include:

1. Operation of CCS facilities associated with producing hydrogen from natural gas may not be constant and may be cut back or turned off depending on electricity price, a CO_2_ emissions price, and more broadly the operating environment, undermining favorable cost estimate that assume constant operation (Budinis et al., 2018; Leung et al., 2014; Martin‐Roberts et al., 2021).
2. Adoption of CCS across the country may vary, requiring either longer pipelines or larger transportation operations. These may, in turn, lower the overall benefits of these projects as compared with colocated operations (Leung et al., 2014).
3. A policy that rewards hydrogen activities but assumes an optimistic or unrealistically low GHG emissions intensity of production and use or does not penalize activities on the basis of actual emissions may lead to lower adoption (Swim & Geiger, 2021).
4. The infrastructure needed to generate and transport both the feedstocks and end products has its own associated GHG emissions and needs to be incorporated in a holistic view of a hydrogen economy (Bauer et al., 2021; Gonzalez Sanchez, 2022).

In addition to these factors, depending on how the natural gas is sourced, and whether existing or new pipes are used, methane leakage from the natural gas system can be a serious problem. Although Robert Howarth and Mark Jacobson (2021) conclude that “the greenhouse gas footprint of blue hydrogen is more than 20% greater than burning natural gas or coal for heat and some 60% greater than burning diesel oil for heat” (Howarth & Jacobson, 2021) others, like Mike Fowler (2021), use different assumptions and conclude

…that blue hydrogen could deliver energy to end‐users with around 80% less greenhouse gas emissions than direct use of natural gas in the near‐term and even less over time … The conclusions of the Howarth and Jacobson paper are driven by a combination of factors including low assumed rates of carbon capture on the natural gas reforming plants that would make blue hydrogen, high assumed energy consumption to operate those carbon capture plants, and an assumption that methane emissions in the natural gas supply chain are both high today and not susceptible to reductions over time. (Fowler, 2021)

In an analysis that assumed the use of the existing natural gas system, Alhamdani et al. (2017) reported that “methane contributed 96% to the total [global warming potential] due to GHG fugitive emissions,” making it the largest single contributor in a simulated SMR process. Several studies have discussed the under‐estimation of methane emissions in official estimates (Alvarez et al., 2018; Rutherford et al., 2021; Zavala‐Araiza et al., 2015). Methane emissions vary by fluid type, geographic region, and the age of the fields (Burns & Grubert, 2021; MacKay et al., 2021), with some identifying the production stage as the largest contributor to emissions (Rutherford et al., 2021), others focusing on tank‐related emissions as the largest contributor (Ravikumar et al., 2020), and some identifying production, gathering and processing as the largest emissions contributors (i.e., “super‐emitters”) (Alvarez et al., 2018).

Several studies note that the magnitude and frequency of methane emissions from super‐ or ultra‐emitters are frequently underreported (Frankenberg et al., 2016; Lauvaux et al., 2022; Rutherford et al., 2021; Sherwin et al., 2024; Zavala‐Araiza et al., 2015). The EPA has assumed normal distributions of emissions along the process steps, when the actual distributions were lognormal, i.e., they include a “heavy tail” of high emitters (Frankenberg et al., 2016). Importantly, even after wells are abandoned, they can continue emitting methane (Boothroyd et al., 2016; Kang et al., 2014). This indicates that full emissions impacts along the lifetime of an oil and gas field are even higher than currently accounted for.

As methane leaks have garnered more attention, the White House Office of Domestic Climate Policy has set out a Methane Emissions Reduction Action Plan (2021), which includes policy proposals with royalties being paid for vented or flared gas, additional rules on transmission pipeline integrity management, establishing “standards for leak detection technologies and practices” and funding within the Infrastructure Investment and Jobs Act for plugging abandoned oil and gas wells (The White House, 2021). Within the Inflation Reduction Act of 2022, incentives were included for methane mitigation, and a tax on oil and gas methane emissions was established (de Oliveira Bredariol et al., 2022). Additionally, on a global stage, the Global Methane Pledge was launched at COP26 in November 2021, where although no countries were assigned targets, and it is a nonbinding pledge, 120 countries pledged to “collectively reduce methane emissions by at least 30% below 2020 levels by 2030” (de Oliveira Bredariol et al., 2022).

### 4.2 Risk from hydrogen leaks

Hydrogen leaks pose two rather different risks: (1) large leaks in confined spaces that could give rise to explosions, and (2) smaller but persistent leaks that can lead to increases in the atmospheric concentration of hydrogen. There is a considerable and well‐established literature on how best to manage the risk of explosion. Awareness of the importance of hydrogen as an indirect greenhouse gas has only begun to spread within the community. In the atmosphere, hydrogen extends the atmospheric lifetime of methane and increases the concentration of water vapor in the stratosphere. Both these processes increase radiated forcing and contribute to climate change (Warwick et al., 2022).

Although the atmospheric lifetime of most conventional air pollutants is just hours or days, the atmospheric lifetime of CO_2_ is many hundreds of years (Dryden et al., 2018). The atmospheric lifetime for methane is roughly 12 years (IPCC, 2021),^1^ and the lifetime of hydrogen is only about two years (Paulot et al., 2021). These dramatically different atmospheric lifetimes complicate the comparison of radiative forcing caused by these three gases.

Because of their very small size and low atomic weight, hydrogen molecules (H_2_) can leak much more readily than molecules of natural gas (largely methane, CH_4_,). Although both natural gas and hydrogen are lighter than air, hydrogen's much lower weight means it does not accumulate as readily in locations such as basements or other enclosed spaces where it can create a risk of explosion. There are well‐developed safety codes and other guidance on avoiding explosions (Fuel Cell & Hydrogen Energy Association, 2023; Ordin, 1997; U.S. Department of Energy, 2023b). Assuming these codes and best practices are followed, explosion risks from the use of hydrogen are minimal.

Regardless of the hydrogen production method, there will almost certainly be some level of hydrogen leaks. As noted above, while hydrogen itself is not a greenhouse gas, Derwent et al. (2001) explain, “hydrogen reacts with tropospheric hydroxyl radicals, emissions of hydrogen to the atmosphere perturb the distributions of methane and ozone, the second and third most important greenhouse gases after CO_2_.” They report an effective GWP_100yr_ ∼5.8. (Derwent et al., 2001). However, because the lifetime of hydrogen in the atmosphere is so short, using a 100‐year integration time is not appropriate. Ocko and Hamburg (2022) estimate the 20‐year GWP for a steady emission of hydrogen to be roughly 30 times that of CO_2_ (Ocko & Hamburg, 2022).

The global warming potential of methane when integrated for 20 years is about 80 (IPCC, 2021). In an analysis for the UK government, Warwick et al. (2022) explain that global warming potential for hydrogen can be deduced by summing the global warming potentials from “perturbations to CH_4_, tropospheric ozone and stratospheric water vapor.” They compute a 100‐year GWP of between 6.4 and 15.3 and a 20‐year GWP of about 32.2 (Warwick et al., 2022).

With technical and management efforts, it may be feasible to keep hydrogen leak rates as low as 0.1% in a professionally operated newly designed tight system for the production of hydrogen from natural gas with CCS (Bond et al., 2011; Schultz et al., 2003). However, for systems that involve multiple actors, assuring that level of performance may prove difficult. Although not much operational data exist in the literature on hydrogen leaks, models and simulations can provide an estimate of what might be expected as a hydrogen economy takes off. Existing studies have broken up risk summaries into three primary stages: production, delivery, and end‐use. Aggregating approximations from such studies, Friedmann et al. (2019) generated models for estimated economy‐wide leakage risks, finding that the leakage rate will be between 2.9% and 5.6% by 2050, with the production stage making up more than half of estimated leakage in both the low‐ and high‐risk scenarios (Friedmann et al., 2019). Assuming a value of $2/kg H_2_, this would not only account for a nonnegligible contribution to global warming but would also represent a yearly $59 billion in value loss within the hydrogen economy.

The U.S. DOE has understood that hydrogen leaks could be a significant issue and recently announced a funding opportunity (U.S. Department of Energy, 2022a) to spend a total of up to $8 million for six to eight studies that focus on the “Development and Validation of Sensor Technology for Monitoring and Measuring of Hydrogen Losses” (U.S. Department of Energy, 2023e).

### 4.3 Risks from NO_x_

Hydrogen can be used as fuel in almost any application in which fossil fuel is combusted to produce heat, in fuel cells and in reciprocating engines and gas turbines (Hydrogen Council, 2023). Although the primary by‐product from these applications is water, if combustion occurs in air, NO_x_—a recognized and regulated air pollutant—can also be produced as a by‐product during high‐temperature combustion (A. Lewis, 2021).

Hydrogen end uses will determine the levels of NO_x_ emissions that may be generated as a result of hydrogen combustion, with fuel cell applications having no NO_x_ contributions, but any application that involves combustion above 750^◦^C will have a direct relationship with the intensity of NO_x_ emissions. However, as with diesel engines, for example, technologies exist to treat exhaust gases to reduce NO_x_ emissions following combustion (Lewis, 2021).

## 5 DISCUSSION AND CONCLUSIONS

This paper has reviewed both the economic and environmental risks of hydrogen as a fuel and energy carrier and evaluated whether and how risks could be mitigated or proactively managed in hydrogen hubs. Below we summarize the major risks and potential hub risk mitigation and management strategies. We conclude by suggesting design principles for early hudrogen hubs and the larger hydrogen ecosystem.

### 5.1 Recapping the major risks

Several of the risks we discuss can be mitigated by starting the development of a hydrogen economy through the use of hydrogen hubs. These include the ability to learn about viable business models and monitor systems on a small scale while relying on new, fit‐for‐purpose infrastructure that is less likely to break or leak. Other risks will still require attention and active management.

Several risks are interrelated. For example, success in managing environmental risks, and the communication of these risk mitigation actions, can reduce the risk that the public may oppose hub development. Despite occasional major explosive events, society has managed to operate other hazardous systems, including the distribution of gasoline and natural gas, with limited public opposition. However, the limited public opposition to these systems should not be taken as a sign of automatic public acceptance of hydrogen systems. For this reason, care to avoid a major event—such as a major leak caused by embrittlement that could trigger wide public concern—will be especially important. Managing these risks will require extensive use of best practices and careful attention and inspection by state and federal regulators, as well as industry associations. In addition, it will require training of emergency personnel on safe hydrogen handling.

The limited scale of a hub can go a long way toward mitigating environmental and safety risks due to the more modest scales of hydrogen storage and transport. In the short term, the volumes produced by most hydrogen hubs will likely only require above‐ground tank storage. However, as volumes increase, producers will likely want to turn to geological storage of the type widely used for natural gas. However, the much lower atomic weight, and much greater propensity to leak, associated with hydrogen mean that more attention must be devoted to assessing the integrity of geologic storage facilities. This is especially true because the level of avoided leakage that must be maintained to serve the economic interests of producers will be substantially higher than the level that should be achieved to prevent contributions to greenhouse warming.

Although hydrogen is not a direct greenhouse gas, when it is released into the atmosphere, it contributes indirectly to greenhouse warming. The community promoting the adoption of hydrogen has been slow to recognize and come to grips with this fact. Constructing hydrogen production and use chains that have very low levels of leakage will be challenging. A failure to address this issue up front could give rise to the need for expensive retrofits of the sort that are now plaguing natural gas—complicated by the fact that unlike natural gas hydrogen leaks cannot be easily detected or imaged. The renewed interest has included considerable discussions with emergency response personnel and a federally sponsored initiative to more accurately monitor hydrogen volumes (U.S. Department of Energy, 2023f).

For similar reasons, hubs may also prove a helpful configuration for managing the economic and environmental risks of CO_2_ capture, transport, and sequestration. Making hydrogen from natural gas with near‐zero GHG emissions is not the only technology that is going to require the use of CCS. To avoid widespread opposition, great care should be taken in the design of reservoirs and in minimizing induced seismicity. Current interagency efforts at the federal level to speed up the process of securing approval of sequestration wells are promising, but the difficulty of overcoming legal and regulatory barriers should not be minimized. Growing interest in delegating the approval process to states is also encouraging, but carries some risk of overenthusiastic approvals, with too little attention paid to issues of liability and long‐term stewardship, giving rise to subsequent environmental and safety problems. A single major event arising from inadequate regulatory oversight could easily cast a shadow across the entire hydrogen hub enterprise.

Risks of adverse impacts on communities and workers could also be mitigated in a hub setting but would still need to be actively monitored and managed. The potential risk of exacerbating local air quality degradation could also be more easily addressed in a hub setting. Combustion of hydrogen in air can result in the creation of NO_x_. Both national EPA and state environmental regulations should be sufficient to address this risk if they are implemented in a systematic and timely manner. After big promises, the risk that local jobs do not materialize could become a potent factor undermining the support of labor unions and other workforce advocacy groups, especially in the face of high levels of public concern about social equity and community impacts. This risk points to the need for early and continued investment in identifying viable career pathways for workers that reflect skill, salary, and other requirements and for augmenting training programs in community colleges and technical schools to ensure workers can move into attractive jobs.

In summary, if managed well, the development of regional hydrogen hubs is a strategy that holds promise to help in the process of decarbonizing the U.S. and global economies. Managed poorly, early hydrogen hubs could seriously impede the subsequent, urgently needed development of alternative clean energy carriers such as hydrogen. Large premature investments could result in stranded capital and lead to policy dead ends (Morgan, 2016). Hubs offer us an opportunity to take a step‐by‐step approach. Adopted intelligently, with careful consideration of the factors discussed above, hydrogen hubs appear to hold considerable potential.

### 5.2 Design principles for early hydrogen hubs and the larger hydrogen ecosystem

Starting with hydrogen hubs has the potential to mitigate, or at least postpone, confronting some of the major risks to hydrogen system development. However, the remaining risks are still formidable and must be managed effectively to avoid false starts and stranded capital. Based on this review and our analysis, below we offer five design principles for early hydrogen hubs, focusing on mitigating risks.

1. Pay early attention to the development of end users.
2. Be proactive about public engagement and addressing social, economic, political, and climate externalities.
3. Streamline regulatory requirements for the necessary infrastructure development, with an eye on the future integration of end users within the larger hydrogen ecosystem.
4. Encourage and, if necessary, facilitate the transparent cooperation and coordination of participants in the hydrogen ecosystem.
5. Ensure the robust operational performance of the chosen design in light of uncertainties.

*Principle 1: Pay early attention to the development of end users*. Here, we recommend comparing the relative cost of displacing CO_2_ from a range of different end uses through conversion to hydrogen. As discussed earlier, this means that in the case of hydrogen produced from natural gas with CCS, electricity production is unlikely to be an optimal first use. However, heavy‐duty vehicle transportation and high‐utilization‐managed fleets stand out for their ability to facilitate centralized refueling while displacing otherwise difficult‐to‐substitute liquid fuels. In contrast, electrification with low‐emission electricity is widely seen as a superior strategy for light‐duty vehicles.

Recognizing the need for a steady supply and demand for a successful hydrogen market, the U.S. federal government included an additional $1 billion in the original hydrogen hub funding opportunity, to encourage what is now termed the “Hydrogen Demand Initiative” (U.S. Department of Energy, 2024b). In addition, federal and state governments should encourage private–public work both on improving current applications and encouraging novel applications of hydrogen.

In this same vein, as these applications are continuously being improved, education of government and business decision‐makers may also drive the adoption and integration of hydrogen across larger swaths of industry.

*Principle 2: Adopt a proactive approach to public engagement and externalities*. Externalities are the impacts of a project that are not captured in its cost or revenue. There is a long history of firms and others developing a project based on a promising new technology, only to then encounter serious pushback. There is, of course, no guarantee that early engagement with the public, and with key stakeholders, will ensure rapid acceptance. However, a failure to engage can often lead to serious difficulties and perhaps even a complete impasse (Armstrong, 2021; Batel, 2020; Chow & Leiringer, 2019; Cohen et al., 2014; Devine‐Wright, 2011; Jager et al., 2019; Renn et al., 2020).

Viewed in isolation, virtually any new technology involves some undesirable or adverse attributes. In framing the introduction of a new technology, such as the use of hydrogen as an energy carrier, it is important to adopt a broad comparative perspective. Prior work, for example on CCS, suggests that when a technology is introduced in the context of alternatives, and trade‐offs are made explicit, public acceptance can grow substantially (Fleishman et al., 2010).

Managing the risks of public opposition is likely to depend on who benefits from hubs. Locating hubs in energy communities that are at risk of losing substantial employment in a clean energy transition could help to offset or even reverse negative workforce effects. Of course, the direct financial costs and rewards of a hub may neglect spillovers to learning or demonstration of new technology, which may be borne by one developer but benefit many future providers. Where such opportunities for spillover and learning are expected to be large, private hub designers may have a strong justification for leveraging public funding. To ensure that these benefits accrue, governments should insist on formal plans and well‐articulated strategies as a condition for providing support—ensuring the current Community Benefits Plans are executed in a way that allows for active engagement with frontline communities (U.S. Department of Energy, 2023a). Continuous, transparent, accessible, and repeated engagement with those members of communities should be expected in every government‐sponsored project. If this condition is met, the government should consider assessing the level of funding required to clear key thresholds for success, rather than sprinkling funds across multiple projects without attention to need. Finally, political externalities—impacts on the timing and degree of stakeholder consensus over whether and how hydrogen hubs scale beyond the regional level—should not be ignored. It will be especially important to monitor impacts in communities as the hydrogen ecosystem grows and new firms and infrastructure may develop outside the purview of the hydrogen hubs. As these peripheral developments would not be subject to the same oversight and requirements as the federally sponsored hydrogen hubs, it will be essential that those participating in the hydrogen ecosystem are expected to maintain similarly high standards in community engagement. Ensuring hubs are designed in a way that minimizes leakage and are located so that they do not exacerbate social inequalities, negative public perceptions or political tensions will be vital to the success of early designs.

*Principle 3: Streamline regulatory requirements for the necessary infrastructure development, with an eye on the future integration of end users within the larger hydrogen ecosystem*. Though the current hydrogen hub designs are geographically separated, as hydrogen volumes increase, pipelines for hydrogen transportation will become the more economically advantageous choice. Understanding the long‐term planning associated with developing such infrastructure projects, state, local, and federal governments should work to streamline the processes.

In addition to the physical infrastructure development, as mentioned in Section 3.3, the development of consistent standards in end‐product definitions (especially as related to carbon‐intensity measurements and purity requirements) will allow firms to integrate hydrogen adoption strategies more accurately into their financial planning—mitigating an economic uncertainty that currently leads to hesitation in hydrogen demand.

*Principle 4: Encourage and, if necessary, facilitate the cooperation and coordination of participants in the hydrogen ecosystem*. As various projects around the United States, both for production and offtake are developing, due to the nascent and competitive nature of the environment, silos may also form between hubs and/or between organizations. However, there is room for sharing lessons learned and certain internal analyses, especially in the context of government entities. Ports, for example, which may be government or publicly owned, could share their assessment methods for integrating hydrogen in maritime operations. Similarly, transit authorities—especially within the context of their own states and the associated policy incentives that they operate under—could share their internal assessments with each other on short‐ and long‐term integration of hydrogen‐powered buses. If the government—either at a federal or state level—leads more transparent cooperation and coordination between these types of entities, the risk of being a first mover in this space would diminish.

*Principle 5: Ensure the robust operational performance of the chosen design in light of uncertainties*. Designs with operational flexibility may have advantages given numerous uncertainties. Developers should consider whether candidate designs perform well regardless of how the numerous uncertainties and risks are ultimately resolved, rather than focusing on net present value alone. Real options are one potential approach to assessing the value of design flexibility under uncertainty (de Neufville, 2003). The uncertainties include related, supporting, and competing technology costs, the success and timing of efforts to address hydrogen and methane leakage rates from storage and transportation infrastructures, possible regulatory complications, and the extent of public policy support. Evaluating vulnerability to unfavorable realizations of these uncertainties will be an essential step in the design process. Far from being a negative exercise, exploring these uncertainties openly in conversations among regional stakeholders can prompt rigorous thinking and evaluation that increase the likelihood that hubs move beyond public relations exercises to pave the way for expanded, more complex, regionally connected hydrogen‐based energy systems.

## Footnotes

1 The final report of WG 1 of IPCC AR6 reports a lifetime for methane of 11.8 ± 1.8.
